# ﻿A split decision: molecular and biogeographical evidence support species-level status of *Anguispira kochi* and *Anguispira occidentalis* (Stylommatophora, Discidae)

**DOI:** 10.3897/zookeys.1261.171098

**Published:** 2025-12-02

**Authors:** Robert G. Forsyth, Annegret Nicolai, Nathaniel F. Shoobs, Reham F. Ali, Rodrigo B. Salvador

**Affiliations:** 1 Department of Natural History, Research and Collections Centre, New Brunswick Museum, Saint John, New Brunswick, Canada New Brunswick Museum Saint John Canada; 2 Living Lab CLEF, Plélan-Le-Grand, France Living Lab CLEF Plélan-Le-Grand France; 3 Station Biologique de Paimpont, Ecobio, Université de Rennes, Rennes, France Université de Rennes Rennes France; 4 Museum of Biological Diversity, Department of Evolution, Ecology and Organismal Biology, The Ohio State University, Columbus, USA The Ohio State University Columbus United States of America; 5 Department of Zoology and Agricultural Nematology, Faculty of Agriculture, Cairo University, Giza, Egypt Cairo University Giza Egypt; 6 Faculty of Organic Agriculture, Heliopolis University for Sustainable Development, Cairo, Egypt Heliopolis University for Sustainable Development Cairo Egypt; 7 Zoology Unit, Finnish Museum of Natural History, University of Helsinki, Helsinki, Finland University of Helsinki Helsinki Finland; 8 Arctic Chronobiology and Physiology Research Group, Department of Arctic and Marine Biology, UiT – The Arctic University of Norway, Tromsø, Norway UiT – The Arctic University of Norway Tromsø Norway

**Keywords:** Banded tigersnail, conservation status, Discoidea, Eupulmonata, molecular phylogenetics, *
Zonodiscus
*

## Abstract

*Anguispira kochi* (L. Pfeiffer, 1846), the banded tigersnail, is a North American member of the family Discidae. It is typically subdivided into two subspecies: *A. kochi kochi* (L. Pfeiffer, 1846) and *A. kochi occidentalis* (E. von Martens, 1882). Genetic data from throughout the distribution of *A. kochi* (sensu lato) was used to produce a phylogenetic analysis and, together with morphological and distributional data, assess the taxonomic status of its subspecies. Evidence for elevating the western subspecies, *A. k. occidentalis*, to the rank of species level is provided and the implications this change has for the conservation status (and future conservation plans) of both species is discussed. A lectotype is designated for *A. occidentalis*. The status of other infraspecific taxa of both *A. kochi* and *A. occidentalis* is discussed, as well as the potential validity of subgenus Zonodiscus Pilsbry, 1948. Finally, the new classification of *Discus marmorensis* H.B. Baker, 1932 as *Anguispira marmorensis* (H.B. Baker, 1932) comb. nov. is proposed.

## ﻿Introduction

The genus *Anguispira* Morse, 1864 (Eupulmonata, Discidae) is a group of rather large terrestrial snails typically inhabiting moist forested areas in both eastern and western North America (Rankin et al. 2021). It contains 13 species according to Turgeon et al. (1998) or 21 species and subspecies according to Schileyko (2002). The current classification of this group largely follows Pilsbry (1948), although Solem (1976) revised some species. Pilsbry (1948) recognised two subgenera, the nominate subgenus and a new subgenus, *Zonodiscus* Pilsbry, 1948, which he differentiated by characters of the shell and genitalia. Nearly all species of the nominate subgenus have an eastern and central North American distribution, with no representatives in the west and only one species in Canada: the type species, *Anguispira alternata* (Say, 1817). On the other hand, Anguispira (Zonodiscus) occurs in both western and eastern North America, but supposedly with a single species, *A. kochi* (L. Pfeiffer, 1846). Among at least all large terrestrial snail species, *A. kochi* is unusual in having remarkably disjunct eastern and western populations in North America; these are separated by a gap of more than 2,000 km. This disjunction is biologically real and not due to insufficient surveys or lack of knowledge (Pilsbry 1948; Hubricht 1985).

Several purported infraspecific taxa, either as varieties or forms, of *A. kochi* have been described at various times (Walker 1906; Clapp 1916; Clench and Banks 1939; Pilsbry 1948) and have importance in the conservation assessment of *A. kochi* should they be evolutionarily significant. Three infraspecific taxa were described by Clapp (1916), including *Pyramidula solitaria mynesites* Clapp, 1916, *P. s. strontiana* Clapp, 1916, and *P. s. roseo-apicata* Clapp, 1916. Pilsbry (1948: 593) considered these as “minor strains” established as “pure races” and treated these taxa as a form (*mynesites*) and two subspecies (*strontiana* and *roseoapicata*) of *A. kochi.*Hubricht (1985) considered all Lake Erie infraspecific taxa to be synonyms of *A. kochi*, although he did not provide his reasons.

Pilsbry (1948) recognised the western *A. kochi occidentalis* (E. von Martens, 1882) as a separate subspecies from the eastern stock, although the distinctions were not made entirely clear, and he wrote that some specimens from the east and west were nearly conchologically identical. The western group was first formally described by Martens (1882) as a variety of *Patula solitaria* (an earlier invalid name for *A. kochi*). Later, *A. kochi eyerdami* Clench & Banks, 1939, was described from Yakima County, Washington State by Clench and Banks (1939). It purportedly differs from *A. k. occidentalis* in having a smaller, darker, and flatter shell. Pilsbry (1948: 597) noted a broad range of colour, relative heights, and sizes that may be phenotypes found throughout the range of *A. kochi occidentalis* and gave *eyerdami* no special recognition (i.e. not a subspecies). However, most recently, Burke (2013) unconvincingly treated *A. k. eyerdami* as a separate subspecies.

Thus, over the years, there have been plenty of new taxa proposed within *A. kochi.* In terrestrial malacology, the use of the subspecies category has often been applied rather loosely for commonplace variation that should not warrant formal recognition (e.g. Coan and Roth 1987). But on the other hand, cryptic and little-known taxa have often been first recognised as infraspecific, only later to be determined to represent species in their own right, either by morphological (e.g. Roth and Miller 1993) or molecular data (e.g. Weaver et al. 2006). Even so, Páll-Gergely et al. (2019) have shown that the ratio of subspecies described per species is positively correlated with morphological complexity, range size, and habitat type in multiple land-snail families, shedding light on some of the biological features that influence taxonomists’ decisions to describe and maintain subspecies rather than species.

In 2017, the conservation status of Canadian populations of *A. kochi* was assessed by the Committee on the Status of Endangered Wildlife in Canada (COSEWIC 2017). Two of the three Lake Erie island taxa—*strontiana* from Middle Sister Island and *roseoapicata* from North Harbour, East Sister, and Middle islands—were deemed not to have any conservation value in their own right (COSEWIC 2017), but the western and eastern populations of *A. kochi* were treated as subspecies, following Pilsbry (1948), and assessed separately. The Western Banded Tigersnail, *A. kochi occidentalis*, was found to be Not at Risk, but the eastern banded tigersnail, *A. kochi kochi*, was assessed as Endangered (COSEWIC 2017). The Canadian population of this eastern subspecies is only present in small, isolated patches of habitat on Middle and Pelee Islands in Lake Erie. Subpopulations from other, smaller islands are probably now extirpated, caused by habitat destruction of over-abundant double-crested cormorants, *Phalacrocorax auritus* (Lesson, 1831), and by human activities (COSEWIC 2017).

*Anguispira kochi* has not had a comparable nationwide assessment in the USA, although NatureServe (2025) provides national and subnational ranks for both the USA and Canada. In the USA, the eastern populations are considered Critically Imperilled (S1) in Michigan and West Virginia, Imperilled (S2) in Kentucky and Tennessee, Vulnerable (S3) in Pennsylvania, and Not Ranked in Illinois, Indiana, Missouri, and Ohio, although state-specific statuses may change following the 2025 update to U.S. State Wildlife Action Plans (Association of Fish & Wildlife Agencies 2025). The populations in Ontario are S1. Nowhere (when ranked) are the eastern populations Apparently Secure or Secure (S4 or S5). The western populations appear to be faring better and are ranked as Secure (S5) in Idaho and Montana, Vulnerable (S3) in Washington and British Columbia, and Not Ranked in Oregon.

NatureServe’s national rank for *A. kochi* is Vulnerable (N3) for Canada and Secure (N5) for the USA, but this is misleading. In both countries, but especially in the USA, the most secure western populations (best S-ranks) appear to counteract the ranks of the less secure eastern populations, resulting in more secure national ranks than would be warranted if the western and eastern subspecies were actually separate species.

Herein, we use genetic data of *A. kochi* from throughout its distribution and assess the taxonomic status of its populations and nominal subspecies. We provide evidence for elevating *A. kochi kochi* and *A. kochi occidentalis* to species level.

## ﻿Materials and methods

The taxonomic status of the two subspecies was assessed through phylogenetic analyses (Bayesian inference) using molecular data from different genetic markers. To that end, we conducted two analyses (see below for parameters): (1) analysis with multiple markers, including the mitochondrial markers COI and 16S, alongside the consecutive nuclear markers 5.8S, ITS2, and 28S. This analysis relied heavily on sequence data of Discoidea generated by our team, part of which is new and part of which is already published (Salvador et al. 2020, 2023); to these, some other published sequences from other authors were added (Clutts 2008; Rankin et al. 2021), obtained from GenBank. See Table 1 for a full list. To that end, we assembled a representative set of *Anguispira* species, considering the sequences available for all markers (Table 1). This includes the type species *Anguispira alternata*, as well as the two problematic species pointed out in the phylogenetic study of Salvador et al. (2023): *Anguispira nimapuna* H.B. Baker, 1932 and *Discus marmorensis* H.B. Baker, 1932. *Gonyodiscus rotundatus* (O.F. Müller, 1774) was chosen as the outgroup, considering the previous phylogenies of Salvador et al. (2020, 2023); sequence data for this species were likewise obtained from GenBank (Dinapoli and Kluss­mann-Kolb 2010). (2) Analysis using only the COI barcoding marker, due to the large availability of COI sequences of *Anguispira* spp. on GenBank (Suppl.material 1). This analysis included all sequences currently available for the taxa of interest (*A. kochi kochi* and *A. kochi occidentalis*), as well as the additional species and outgroup from the multi-marker analysis.

**Table 1.** Species used for the multi-marker phylogenetic analysis, including GenBank accession numbers, collection localities, and data sources.

Species	COI	16S	5.8S+ITS2+28S	Location	Source
*Anguispira alabama* (Clapp, 1920)	–	ON749857	ON749850	USA, AL, Jackson	Salvador et al. 2023
*Anguispira alternata* (Say, 1817)	MN792584	MN756711	MN782441	USA, IL, Sangamon	Salvador et al. 2020
*Anguispira alternata* (Say, 1817)	MN792583	MN756710	MN782440	Canada, ON	Salvador et al. 2020
*Anguispira cumberlandiana* (I. Lea, 1840)	MW543329	MW544210	–	USA, TN, Battle Branch (N of Kimball)	Clutts 2008; Rankin et al. 2021
*Anguispira fergusoni* (Bland, 1862)	MW543321	MW544221	–	USA, DE, Blackbird State Forest	Clutts 2008; Rankin et al. 2021
*Anguispira jessica* Kutchka, 1938	MN792585	MN756712	MN782442	USA, NC, Macon	Salvador et al. 2020
*Anguispira nimapuna* H.B. Baker, 1932	MN792588	MN756715	MN782445	USA, ID, Lowell	Salvador et al. 2020
*Anguispira nimapuna* H.B. Baker, 1932	MW532808	MW525438	–	USA, ID, Nez Perce National Forest	Rankin et al. 2021
*Anguispira picta* (G. H. Clapp, 1920)	MW543311	MW544248	–	USA, TN, Buck Creek Cove	Clutts 2008; Rankin et al. 2021
*Anguispira strongyloides* (L. Pfeiffer, 1855)	MN792589	MN756716	MN782446	USA, AL, Stevenson / FL	Salvador et al. 2020
*Anguispira kochi occidentalis* (E. von Martens, 1882)	MW532916	MW533672	–	USA, ID, Seven Devils Mountains	Rankin et al. 2021
*Anguispira kochi occidentalis* (E. von Martens, 1882)	MW532935	MW533691	–	USA, MT, Mineral Co., McGee Creek	Rankin et al. 2021
*Anguispira kochi occidentalis* (E. von Martens, 1882)	MN792586	MN756713	MN782443	Canada, BC, Bear Creek	Salvador et al. 2020
*Anguispira kochi occidentalis* (E. von Martens, 1882)	ON751968	ON749858	ON749851	Canada, BC, Dodge Creek	Salvador et al. 2023
*Anguispira kochi kochi* (L. Pfeiffer, 1846)	MW543392	MW544259	–	USA, OH, Ottawa Co., Green Island	Rankin et al. 2021
*Anguispira kochi kochi* (L. Pfeiffer, 1846)	PV365190	PV364846	–	USA, OH, Delaware Co., Scioto River, Bellepoint	This study (voucher OSUM -IZG-4959.91)
*Anguispira kochi kochi* (L. Pfeiffer, 1846)	PV365191	PV364847	–	USA, OH, Erie Co., Kelleys Island	This study (voucher OSUM-IZG-15891.2)
*Anguispira kochi kochi* (L. Pfeiffer, 1846)	PV365192	PV364848	–	USA, OH, Ottawa Co., Gibraltar Island	This study (voucher OSUM -IZG-46225.1)
*Anguispira kochi kochi* (L. Pfeiffer, 1846)	MN792587	MN756714	MN782444	USA, IL, Brown Co.	Salvador et al. 2020
*Discus marmorensis* H.B. Baker, 1932	MW543387	MW544270	ON749853	USA, ID, Slate Creek Road / Lucile	Rankin et al. 2021; Salvador et al. 2023
*Gonyodiscus rotundatus* (O.F. Müller, 1774)	FJ917285	FJ917265	FJ917240	Germany, Frankfurt am Main	Dinapoli and Klussmann-Kolb 2010

### ﻿Abbreviations

The following acronyms are used throughout the text for natural history collections: **CM**: Carnegie Museum of Natural History (Pittsburgh, PA, USA); **DMNH**: Delaware Museum of Nature and Science (Wilmington, DE, USA); **MCZ**: Museum of Comparative Zoology, Harvard University (Cambridge, MA, USA); **NBM**: New Brunswick Museum (Saint John, NB, Canada); **NHMUK**: Natural History Museum (London, UK); **Lc**: Mollusk Collection, Zoological Museum, Moscow Lomonosov State University, Moscow, Russia; **LIEV**: Mollusk Collection, Oberösterreichisches Landesmuseum (Linz, Austria); **OSUM**: Gastropod Collection, Division of Invertebrate Zoology, Museum of Biological Diversity, The Ohio State University (Columbus, OH, USA); **USNM**: Smithsonian National Museum of Natural History (Washington, DC, USA); **ZMB**: Museum für Naturkunde (Berlin, Germany).

### ﻿DNA extraction and amplification

Given the poor representation of *A. kochi kochi* in GenBank, we sequenced additional specimens from this species, vouchers of which are housed in the OSUM collection (Table 1). A small tissue clip was obtained from the foot of each voucher specimen and DNA extraction followed the standard protocol of the QIAGEN DNEasy^®^ Blood & Tissue Kit, adding one repetition of the final step to increase yield. For the COI marker, the invertebrate primers LCO/HCO of Folmer et al. (1994) were used, while for 16S, primers 16SarL/16SbrH were used (Simon et al. 1994). The protocol for PCR amplification consisted of an initial denaturation step of 3 min at 96 °C, followed by 35 cycles of denaturation at 95 °C (30 s), annealing at either 48 °C (COI) or 51 °C (16S) (1 min), and extension at 72 °C (2 min); final extension step of 5 min at 72 °C. The success of PCR was assessed via agarose-gel electrophoresis. PCR products were cleaned using ExoSAP-IT™ (Affymetrix Inc.) and samples were prepared and sent to Macrogen Europe (Amsterdam, The Netherlands) for Sanger sequencing. The sequences were *de novo* assembled and quality-checked (via Phred scores) in Geneious Prime (v. 2025, Biomatters Ltd) and then uploaded to GenBank (Table 1).

### ﻿Phylogenetic analyses

Alignment of the sequences was conducted via the MUSCLE plugin in Geneious Prime (Edgar 2004). The resulting alignments for each marker were visually proofed for inconsistencies in Geneious. Alignments of the 16S and 5.8S+ITS2+28S markers were run through Gblocks (Talavera and Castresana 2007), using the least restrictive settings, to eliminate poorly aligned or data-deficient positions that could introduce noise into the analysis. The final alignments contained 644 bp (COI), 389 bp (16S), and 1279 bp (5.8S+ITS2+28S).

All alignments were then concatenated for a single multi-marker Bayesian inference phylogenetic analysis. This was performed using MrBayes (v. 3.2.7, Ronquist et al. 2012) via the CIPRES Science Gateway (v. 3.3, Miller et al. 2015). Two concurrent analyses with four Markov chains each were run for 50 million generations, with the first 20% discarded as “burn-in”. The default priors were used, with nst = 6 (GTR), rates = invgamma, temperature parameter = 0.1; substitution model parameters were unlinked across the markers (COI, 16S, and 5.8S+ITS+28S). MCMC convergence was assessed by examining the standard deviation of split frequencies (<0.001), potential scale reduction factor (PSRF = 1.0), as well as trace plots (Ronquist et al. 2009).

The COI-only analysis was done with the same settings, except for the number of generations (40 million). Compared to the multi-marker analysis, this analysis included 134 additional sequences of *A. k. kochi* and *A. k. occidentalis* (Suppl. material 1).

### ﻿Geographical distribution

Data were downloaded from Global Biodiversity Information Facility (GBIF) and cleaned of obviously erroneous records. For records without geographical coordinates, Google Earth, Google Maps, or Bing Maps were used to find, where possible, approximate geopositions. When the coordinates were too generalised (e.g. centre of a state) or when locality data were too vague to determine coordinates, records were discarded. Additional data were added to the GBIF data from the following sources: OSUM database; RGF database of collections and observations; Ovaska et al. (2020).

The resulting dataset included 971 unique data points (Suppl. material 2), which were mapped in QGIS v. 3.38.1. The national and subnational borders were downloaded as .shp files from Natural Earth v. 5.1.1. The map was later modified in Adobe Illustrator v. 29.5.1.

### ﻿Photography

As images of shells were received from multiple institutions with different photography equipment, colours and white balances were corrected in Adobe Lightroom Classic CC v. 14.4 using calibration cards included in each raw image. Specimen photographs from NHMUK and CM, as well as Canadian specimens in Figure 4, did not include colour calibration cards, so white balance was manually adjusted. Figures of specimens were combined in Adobe Photoshop v. 26.11.0.

## ﻿Results

**Figure 1.**
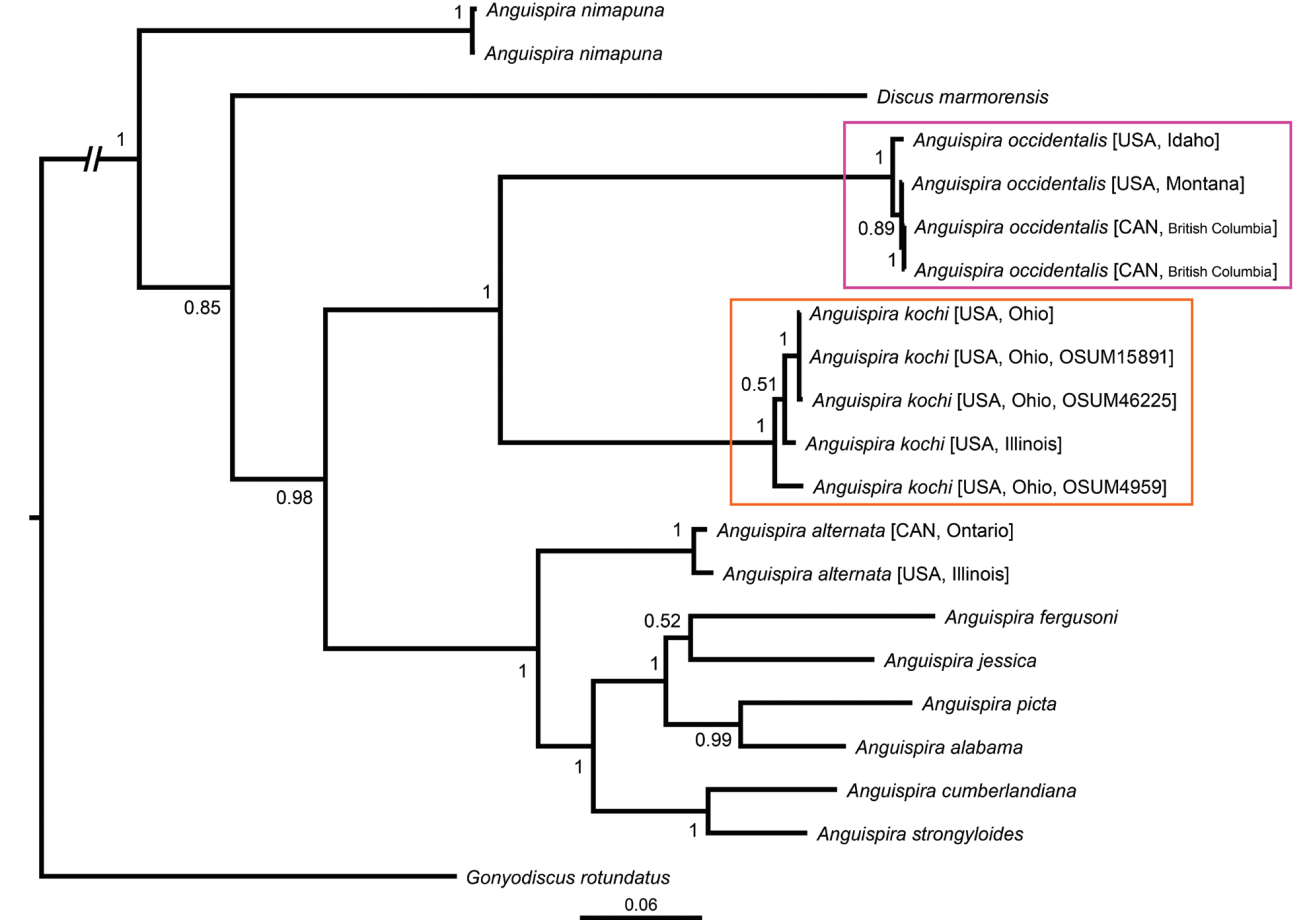
Bayesian inference phylogenetic tree (50% majority-rule consensus) based on the concatenated nuclear and mitochondrial markers. The clades formed by *Anguispira kochi* and *Anguispira occidentalis* are marked in different colours. Posterior probabilities are shown on nodes; the scale bar represents substitutions per site.

The multi-marker phylogenetic analysis, i.e. containing both nuclear and mitochondrial markers, included a total of 2312 bp (1033 mitochondrial and 1279 nuclear) and 21 terminals. The resulting tree largely agrees with former studies (Salvador et al. 2020, 2023; Rankin et al. 2021). In it (Fig. 1), *Anguispira* is a well-supported clade (posterior probability PP = 1), with *A. nimapuna* being the sister taxon to a clade (low support, PP = 0.85) containing all other species. After the branching of *Discus marmorensis*, there is a strongly supported (PP = 0.98) clade containing two sister groups: the first (PP = 1) includes *A. kochi kochi* and *A. kochi occidentalis*; the second (PP = 1) includes the remaining *Anguispira* spp., including the type species *A. alternata*. Notably, *A. kochi kochi* and *A. kochi occidentalis* each form monophyletic clades with strong support (PP = 1). The genetic distance between these two purported subspecies clades is similar or larger than between most other *Anguispira* spp. pairs (Fig. 1). While specimens within each of the *kochi* and *occidentalis* clades have 97–100% identity of their COI sequences, the two clades have only 81–82% identity between themselves. Level of identity between the other Anguispira species range roughly from 75% to 82%.

The COI-only tree (Suppl. material 3) included a total of 645 bp and 154 terminals. Again, the monophyly of both *A. kochi kochi* (PP = 1) and *A. kochi occidentalis* (PP = 1) can be observed. However, this tree shows more geographically delineated clades, with *A. kochi kochi* as the sister of all other *Anguispira* spp., and *A. kochi occidentalis* nested well within the crown group, being the sister species to *Discus marmorensis*.

**Figure 2.**
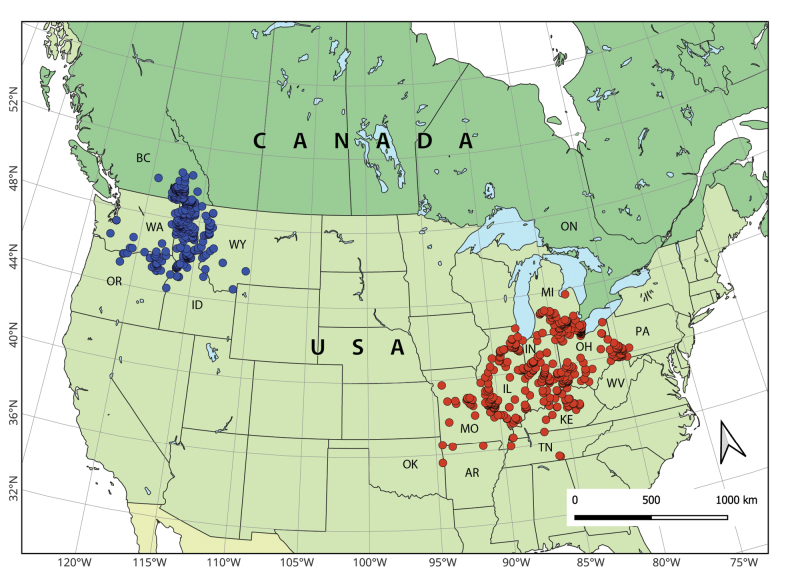
Map showing the geographical distribution of *Anguispira kochi* (red dots) and *Anguispira occidentalis* (blue dots). Occurrence data used to map the species are available in Suppl. material 2.

The distribution map (Fig. 2) shows, as expected, two unambiguously isolated clusters, which agrees with Pilsbry’s (1948) understanding of the range. *Anguispira kochi occidentalis* is restricted to the northwestern United States and southeastern British Columbia, Canada. *Anguispira kochi kochi* is distributed along several states in the eastern portion of the USA, being mostly restricted to the eastern states of the Midwest but also occurring in a few other areas. Additionally, several nominal subspecies of *A. kochi* are known from the islands of western Lake Erie in Ohio, USA and Ontario, Canada.

We also took the opportunity to reassess the problematic records of our species of interest coming from unexpected locations (i.e. Utah and Colorado). To that end, we tracked down the voucher specimens in museum collections and obtained photographs and further information from museum staff. Specimens from lot CM 102529 were mistakenly assigned to Utah, but are in fact from the Bitterroot Mountains, northern Idaho (T. Pearce pers. comm. 2025). Specimens USNM 853254 and USNM 853255 from Colorado are not *Anguispira* and have been reidentified as Oreohelix cf. strigosa (A.A. Gould, 1846). Schileyko (2002: 1060, fig. 1384) figured the shell and reproductive anatomy of specimens he identified as *A. kochi kochi* (Lc-20459 and Lc-20442), from “Weeping Point, Zion Canyon, [Utah]”, likely from the same original lot as LIEV 2011/14/7095 (all are from the C. Frank-Fellner Collection). We were unable to examine Schileyko’s specimens but conclude that they are all likely Oreohelix cf. strigosa based on the location: “Weeping Point, Zion Canyon” is likely the same place as “Weeping Rock”, a famous landmark in Zion National Park where *O. strigosa* is known to occur (Gregg 1940).

## ﻿Discussion

According to both the genetic data (both mitochondrial and nuclear) and the geographical distribution, we can consider *Anguispira kochi* and *A. occidentalis* as distinct species. Results from the phylogenetic analysis agree with previous studies (Salvador et al. 2020, 2023; Rankin et al. 2021). The genetic distance between *A. kochi* and *A. occidentalis* is similar or greater than that observed between most species pairs in the genus in the multi-marker phylogeny (Fig. 1). That is also seen in the pairwise identity values between clades. Furthermore, the geographical ranges of *A. kochi* and *A. occidentalis* are entirely isolated, with a large gap (>2,00 km) between them (Fig. 2). When taken together, this evidence indicates that the split between *A. kochi* and *A. occidentalis* is an old one.

The COI tree, with a large number of terminals, also argues in favour of this interpretation. This mitochondrial marker shows that there is a greater similarity between *A. occidentalis* and other *Anguispira* spp. than with *A. kochi* (Suppl. material 3). That could be expected in a tree consisting solely of COI, as it is observed that mitochondrial lineages of these slow-dispersing animals often form geographical clusters (e.g. Nekola et al. 2015, 2025b). Thus, such a pattern in mitochondrial markers is usually interpreted as evidence of mitochondrial introgression taking place between sympatric or parapatric congeners (e.g. Nekola et al. 2025a).

**Figure 3.**
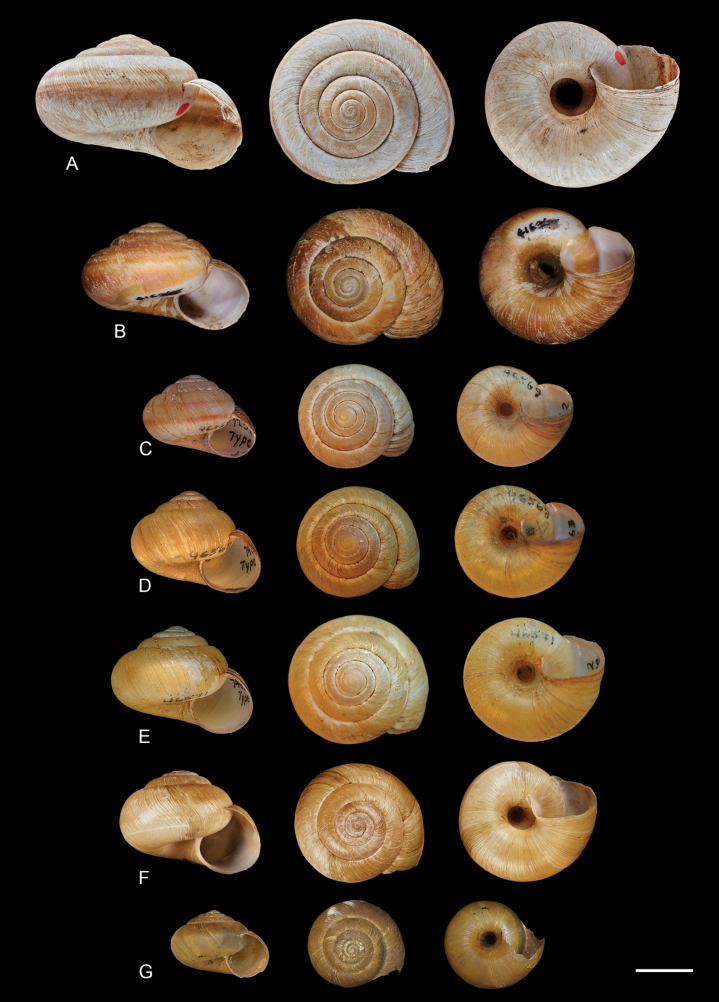
Primary type specimens of nominal subspecies of *Anguispira kochi*, all to scale. Under the new classification scheme proposed in this paper, *A. kochi* (**A–E**) and *A. occidentalis* (**F, G**). **A.** Lectotype of *Helix kochi* L. Pfeiffer, 1846, NHMUK 20250002; **B.** Lectotype of A. kochi var. aperta Pilsbry, 1948, ANSP 166513; **C.** Holotype of Pyramidula solitaria var. mynesites Clapp, 1916, CM 46569; **D.** Holotype of P. solitaria var. roseo-apicata Clapp, 1916, CM 46568; **E.** Holotype of P. solitaria var. strontiana Clapp, 1916, CM 46571; **F.** Lectotype of H. solitaria var. occidentalis; E. von Martens, 1882, ZMB 34978a; **G.** Holotype of *A. kochi eyerdami* Clench & Banks, 1939, MCZ 100491. Scale bar: 20 mm.

**Figure 4.**
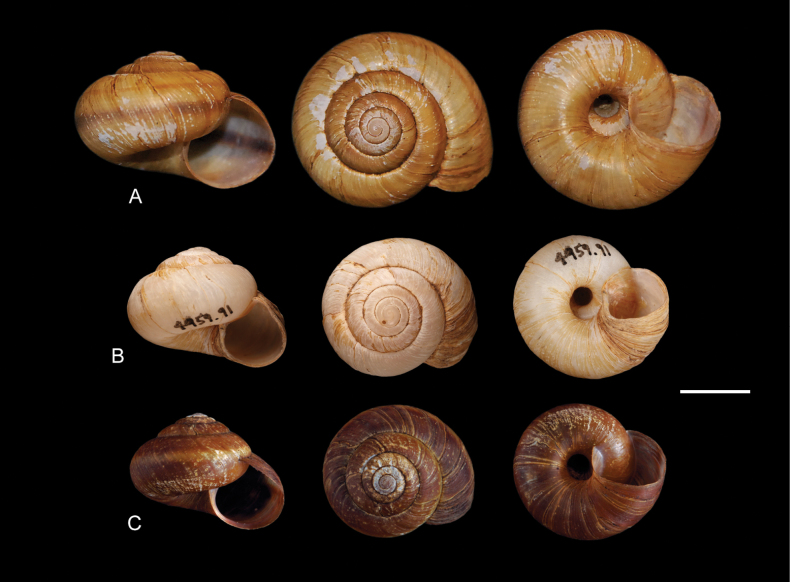
Representative *Anguispira* shells. **A.***A. kochi* from Fish Point, Pelee Island, Essex Co., Ontario, Canada, NBM-MO-011670; **B.** “Albino” *A. kochi* from Bellepoint, Scioto River, Delaware Co., Ohio, USA, OSUM-IZG-4959.91; **C.***A. occidentalis* from Grohman Narrows, Central Kootenay Regional District, British Columbia, Canada, NBM-MO-011671. Scale bar: 20 mm.

Overall, the two species have shells of similar shape (Figs 3, 4), being discoid-conical to subglobose (varying in spire height and overall globosity of the shell), with 5–5½ whorls, a deeply incised suture, marked sinuous axial striation, a simple lip only slightly thickened in adults, and an open umbilicus. Shell colour varies from lighter to darker tones of brown, with two dark spiral bands (which can be externally invisible but still visible inside the aperture). Shell size averages 2.0–2.5 cm in width, with larger specimens reaching up to 3 cm (Clapp 1916; Forsyth 2004; Grimm et al. 2009; COSEWIC 2017). The animal’s head and tentacles are grey, and the foot is orange-red to brown. When disturbed, they produce a slightly orange mucus that fluoresces under UV light (Dourson and West Virginia DNR 2015; COSEWIC 2017).

Typically, *A. occidentalis* has a darker shade of brown as base colour, the spiral bands are less conspicuous, and often the region between the spiral bands seems lighter. On the other hand, *A. kochi* typically has a lighter base colour that contrasts more with the dark spiral bands. This distinction in shell colour is the most straightforward manner to diagnose most specimens (besides geographical provenance, of course), but as Pilsbry (1948) noted, there is variation in both species, and some paler-shelled *A. occidentalis* can be very similar to *A. kochi*. In general, *A. kochi* appears to be the most variable in shell morphology, varying from coarsely striate to nearly smooth, and from bandless and straw-coloured to brown and banded (COSEWIC 2017).

*Anguispira* species are detritivores, feeding on decaying plant material, fungi and lichens on logs or wood (Ali pers. obs.), but they can differ in their habitats. As expected from their distinct biogeographical provinces, *A. kochi* and *A. occidentalis* show differences in habitat use. *Anguispira kochi* has been recorded in ravines and upland forests, being a typical species of old forests and seldom found in second-growth areas (even in thick ones) (Hubricht 1985); they prefer a thick covering of leaf litter and limestone terrain (Pilsbry 1948; Dobbyn and Hoare 2009). *Anguispira occidentalis* is typically found in riparian habitats along rivers, streams, and creeks in mixed-wood or deciduous forests, frequently in the leaf-litter layer or under coarse woody debris (Forsyth 2004; Ovaska et al. 2020).

### ﻿Infraspecific taxa

*Anguispira kochi* was described as *Helix kochi* L. Pfeiffer, 1846 from an unknown locality in the United States (Pfeiffer 1846). Its type locality was subsequently restricted to Cincinnati, Ohio, by Pilsbry (1948: 592), who also pinpointed the type specimen and, in doing so, designated the lectotype according to ICZN Article 74.6 (lectotype NHMUK 20250002; Fig. 3A). *Helix solitaria* Say, 1821 (non *Helix solitaria* Poiret, 1801), described from Lower Missouri (Say 1821), is a synonym of *A. kochi*.

Several subspecies and varieties of *A. kochi* have been described throughout the decades (Fig. 3B–G). Below, we present an overview of these infraspecific taxa and their current statuses.

*Anguispira occidentalis* was described as Patula solitaria var. occidentalis E. von Martens, 1882 (ZMB 34978, 3 syntypes). Its type locality was defined as “Crossing bei den Thompson-Fällen am Clarke’s Fork (Columbia)” (Martens 1882); Thompson Falls is a city located along the Clark Fork of the Columbia River in present-day Montana, USA. As demonstrated above, this taxon can be recognised as a distinct species, *A. occidentalis*. Considering the syntype series and the original description, we designate here specimen ZMB 34978a (Fig. 3F) as the lectotype. The remaining two paralectotypes are grouped in lot ZMB 34978b.

Pyramidula solitaria var. albina Walker, 1906 (see also Binney 1885; Clapp 1916), as expected from its name, refers to an albino form from Kent County, Michigan. “Albinos” (both albino and leucistic individuals) are common in almost all land-snail species for which many specimens are known. Thus, albino varieties and forms are currently understood as phenotypic variations and not accepted as valid infraspecific taxa (e.g. Salvador et al. 2025). We sequenced an “albino” specimen for this project (OSUM-IZG-4959.91; Figs 1, 4B), which has an off-white, unbanded shell and a normally pigmented body, and thus, the variety *albina* can be considered a junior synonym of nominate *kochi*.

Clapp (1916) described three varieties from islands in Lake Erie. Pyramidula solitaria var. strontiana Clapp, 1916 (holotype CM 46571, paratypes CM 71680 and CM 102546; Fig. 3E) was described as a uniformly “straw-colored” shell without bands, marginally smaller than mainland shells (Clapp 1916). Its type locality is Green Island (at one time known as Strontian Island), Ohio, but it was also reported from Middle Sister Island (Clapp 1916). The second variety, Pyramidula solitaria var. roseo-apicata Clapp, 1916 (holotype CM 46568, paratypes CM 71616, CM 102570, and FMNH 60993; Fig. 3D), was described as having an even smaller shell, mostly lacking bands, with an elevated spire and pink apical whorls (Clapp 1916). Its type locality is North Harbour Island, Ontario (Clapp 1916). The final variety is Pyramidula solitaria var. mynesites Clapp, 1916 (holotype CM 46569, paratypes CM 46570, CM 71621, and MCZ 51381; Fig. 3C), which was defined as being even smaller, with the shells having two bands and having a pink apex (Clapp 1916). Its type locality is Mouse Island, Ohio (Clapp 1916).

Intraspecific variation in shell size, colour, and banding is well known across species and families of land snails. In similar latitudes, some notable examples are the European *Cepaea nemoralis* (Linnaeus, 1758), *Cepaea hortensis* (O.F. Müller, 1774), and *Arianta arbustorum* (Linnaeus, 1758) (Helicidae) from Europe. Often, small and isolated populations will have slightly different phenotypes than an average representative of the species and the decision to recognise it as a subspecies can become quite arbitrary (Páll-Gergely et al. 2019). In our analyses, we were able to include sequences of specimens from Gibraltar, Green (type locality of *strontiana*), Kelleys, Middle, and Pelee islands in Lake Erie (Table 1, Suppl. material 1). Furthermore, the genetic distance between them, measured through the COI barcoding marker, is negligible (Suppl. material 3). Thus, we can consider all these varieties as junior synonyms of nominate *kochi*.

*Anguispira kochi eyerdami* Clench & Banks, 1939 (holotype MCZ 100491, paratypes MCZ 100492, ANSP 177574, DMNH 149346; Fig. 3G) has its type locality as a “slope near mountaintop, Horse Haven Hills, Yakima Indian Reservation, Washington. 75 mi. from Satus Creek and 10 mi. S of Alfalfa, Washington” (Clench and Banks 1939). Considering our split of *A. kochi* and *A. occidentalis*, this would thus be a subspecies of the latter. Clench and Banks (1939) defined this subspecies as having a smaller and darker shell, with a more depressed spire than nominate *occidentalis*. Nevertheless, this type of variation in shell size, shape, and colour is observed throughout the species’ range (e.g. Pils­bry 1948; iNaturalist records), and it is expected that some populations could have a higher proportion of the *eyerdami* morph. There were no available sequences belonging to topotypes (the closest is MW532917, from ca 150 km from the type locality; see Rankin et al. 2021 and our Suppl. material 1), but no meaningful genetic distances were observed within specimens of *A. occidentalis* beyond expected geographical clusters (Suppl. material 3). Thus, like the island varieties of *A. kochi* above, for now we maintain *A. occidentalis eyerdami* a junior synonym of *A. occidentalis*.

The last infraspecific taxon described was Anguispira (Zonodiscus) kochi f. aperta Pilsbry, 1948 (lectotype ANSP 166513, paralectotypes ANSP 416316; Fig. 3B), from “Spring Mill State Park, Lawrence Co., Indiana”, defined as being of normal size and having a larger umbilicus (Pilsbry 1948). Notably, Pilsbry (1948) described this as a form, not a subspecies, but such a minimal difference in shell shape hardly justifies a new name. This form is considered synonymous with nominate *A. kochi*.

### ﻿The subgenus Zonodiscus

The subgenus Zonodiscus Pilsbry, 1948 was described based on conchological and anatomical characters: a shell with two dark spiral bands, not always visible or present; a longer vagina than *A. alternata* (the type species of *Anguispira* proper); an enlarged vas deferens; and a smaller pilaster in the penis cavity (in comparison to *A. alternata*) (Pilsbry 1948). Pilsbry (1948) allocated only *A. kochi* within this subgenus, although at that point *A. occidentalis* was considered a subspecies of *A. kochi*.

Our results strongly support *A. kochi* + *A. occidentalis* as a distinct clade from *Anguispira* sensu stricto, with a clear division and large distance between them (Fig. 1), echoing the results of Salvador et al. (2020, 2023). Thus, this could indicate that the subgenus Zonodiscus is a useful taxon. Nevertheless, *Discus marmorensis* and *Anguispira nimapuna* fall outside both the putative *Zonodiscus* clade and the clade comprising *Anguispira* sensu stricto (Fig. 1). Thus, if we recognized *Zonodiscus* as valid, both *Discus marmorensis* and *Anguispira nimapuna* would need a subgeneric name of their own, which is counterproductive. A phylogeny containing representatives of all *Anguispira* species is needed to assess whether *Zonodiscus* could be considered a separate genus and which species would belong in it.

### ﻿*Discus marmorensis*

Although not being a target of this study, it is worthwhile discussing *Discus marmorensis*. This species is nested within *Anguispira* in our trees (Fig. 1; Suppl. material 3). Salvador et al. (2023) had already noted that *D. marmorensis* was not related to *Discus* proper, although their phylogeny did not support a strong relationship with *Anguispira* either. Those authors noted the conchological similarities between *D. marmorensis* and *A. nimapuna*, which are more “*Discus*-like” in shell shape rather than “proper” *Anguispira*. In light of our results, we transfer that species to *Anguispira*, resulting in the new combination *Anguispira marmorensis* (H.B. Baker, 1932), comb. nov.

### ﻿The decline of *A. kochi* populations

Malacologists have long considered *A. kochi* a species on the decline. Pilsbry (1948), citing Goodrich, considered the species a specialist of true old-growth forests, only ever incidentally being found in even mature secondary growth stands. La Rocque (1970: 674), in his monograph on the Pleistocene Mollusca of Ohio, considered the abundance of dead shell assemblages of *A. kochi* in areas devoid of living populations to be evidence of widespread decline. Taylor (1982) indicated he had never observed *A. kochi* in his home state of West Virginia, and reiterated Pilsbry and La Rocque’s position that *A. kochi* s. s. was a rare and declining species restricted to true old-growth forests. While (often unstable) populations persist on some of the Lake Erie islands of the United States and Canada, many historic mainland populations have been extirpated during the last half century (Dourson and West Virginia DNR 2015; COSEWIC 2017). To our knowledge, in the US states of Ohio, Michigan, and Pennsylvania, *A. kochi* has been observed alive only once outside of the Lake Erie islands since the mid 1970s—a recent live observation from Hueston Woods State Park, a small patch of old growth forest in Preble County, OH, USA (Bennett 2025). There have been no recorded observations, live or dead, from West Sister Island, Kelleys Island, Green Island, Mouse Island, North Bass Island, or Middle Bass Island since 1990 or earlier.

Part of this apparent decline may be due to a lack of concerted search effort for the species in the United States. Branson (1984) questioned the view that *A. kochi* was genuinely rare, or an obligate resident of pristine old growth forest, as Pilsbry (1948), La Rocque (1970), and Taylor (1982) maintained. Instead, he proposed that *A. kochi* is less frequently encountered than other land-snail species due to its tendency to burrow deeply in its preferred substrate (loose, moist, humus-rich soil), and described many relatively disturbed sites near Frankfort, KY, USA, bearing abundant populations as examples (Branson 1984). However, Dourson (2010) and Dourson and West Virginia DNR (2015) reported that the species has suffered significant declines in Kentucky and West Virginia primarily due to habitat loss. While it is undoubtedly true that *A. kochi* is under-surveyed for much of its geographical range, it remains the case that it is a large-bodied, colourful species that is frequently encountered by amateurs in certain areas (e.g. iNaturalist records from the Lake Erie islands). In Canada, nearly all historical sites of *A. kochi* have had targeted surveys for its assessment by COSEWIC (2017). However, finding only shells would not be enough to confirm the presence of the species, as shell numbers do not seem to be correlated to the abundance of live individuals of this species (La Rocque 1970; Nicolai pers. obs.). It seems reasonable to assume that the relative rarity of live records in museum and iNaturalist data overall is a reflection of genuine decline. However, targeted surveys are necessary to properly assess the reality and extent of such decline, particularly in an area-by-area basis, as this information should also be useful to authorities in each state.

## ﻿Conclusions

Here, in agreement with previous results (Salvador et al. 2020, 2023; Rankin et al. 2021), we argued for treating *Anguispira occidentalis* as a separate species from *A. kochi*, considering the large genetic and geographical distance between these two taxa. Such a pattern of eastern versus western species vicariance in North America is known from other clades and is considered as evidence in favour of a continuous ancient distribution, with subsequent vicariance into two separate populations (and later species) due to an increase in aridity in the interior of the continent during the Tertiary (Emberton 1994; Emberton and Roth 1994).

Clarification of the systematics of these two former subspecies has significant conservation implications. While both former subspecies are considered Species at Risk in Canada, and *A. kochi* s. s. is legally protected in several US states, it is notably unprotected in Ohio, where the majority of the extant populations are found. Under the Canadian federal Species at Risk Act, reassessments are made every 10 years, and although COSEWIC (2017) was right in assessing the two subspecies separately, as “designable units”, knowing that these biological entities are in fact different species clarifies future assessments. For example, the significant genetic divergence of these sister taxa means that there is no possibility of genetic rescue of *A. kochi* from *A. occidentalis* or vice versa.

Elevating *A. occidentalis* to species rank also means that the U.S. NatureServe’s national rank for *A. kochi* must be revised to accurately reflect the fact that it is restricted to eastern North America. NatureServe’s national ranks for *A. kochi* should no longer be subsidised by the more secure *A. occidentalis* populations in the west, and new targeted surveys focusing on the former are necessary. We hope that this contribution encourages the research and survey work that will be necessary to adequately assess the population status of these poorly known snails.

## ﻿Additional information

### Use of AI

No use of AI was reported.

### Author ORCIDs

Robert G. Forsyth https://orcid.org/0000-0002-9637-0158

Annegret Nicolai https://orcid.org/0000-0001-7099-5603

Nathaniel F. Shoobs https://orcid.org/0000-0002-5272-6255

Reham F. Ali https://orcid.org/0000-0003-3368-8107

Rodrigo B. Salvador https://orcid.org/0000-0002-4238-2276

### Data availability

All data that support the findings of this study are available in the main text or Supplementary Information.

## Supplementary materials

Supplementary material 1 Sequences used for the COI-only phylogenetic analysis, with information on their GenBank accession numbers, the locality where the specimens were collected, and the source of the data Data type xlsx https://binary.pensoft.net/file/1482332This dataset is made available under the Open Database License (http://opendatacommons.org/licenses/odbl/1.0/). The Open Database License (ODbL) is a license agreement intended to allow users to freely share, modify, and use this Dataset while maintaining this same freedom for others, provided that the original source and author(s) are credited.Robert G. Forsyth, Annegret Nicolai, Nathaniel F. Shoobs, Reham F. Ali, Rodrigo B. Salvador

Supplementary material 2 Occurrence data used to produce the map of *Anguispira kochi* and *A. occidentalis* (Fig. 2) Data type xlsx https://binary.pensoft.net/file/1482333This dataset is made available under the Open Database License (http://opendatacommons.org/licenses/odbl/1.0/). The Open Database License (ODbL) is a license agreement intended to allow users to freely share, modify, and use this Dataset while maintaining this same freedom for others, provided that the original source and author(s) are credited.Robert G. Forsyth, Annegret Nicolai, Nathaniel F. Shoobs, Reham F. Ali, Rodrigo B. Salvador

Supplementary material 3 Bayesian inference phylogenetic tree (50% majority-rule consensus) based on the COI barcoding marker Data type pdf Explanation note Posterior probabilities are shown on nodes; the scale bar represents substitutions per site. https://binary.pensoft.net/file/1482334This dataset is made available under the Open Database License (http://opendatacommons.org/licenses/odbl/1.0/). The Open Database License (ODbL) is a license agreement intended to allow users to freely share, modify, and use this Dataset while maintaining this same freedom for others, provided that the original source and author(s) are credited.Robert G. Forsyth, Annegret Nicolai, Nathaniel F. Shoobs, Reham F. Ali, Rodrigo B. Salvador
