# Supplementary figures and images for: ﻿A split decision: molecular and biogeographical evidence support species-level status of Anguispira kochi and Anguispira occidentalis (Stylommatophora, Discidae)
Source: Zookeys. 2025 Dec 2;1261:241–60. doi: 10.3897/zookeys.1261.171098 (PMC12690380; doi:10.3897/zookeys.1261.171098)

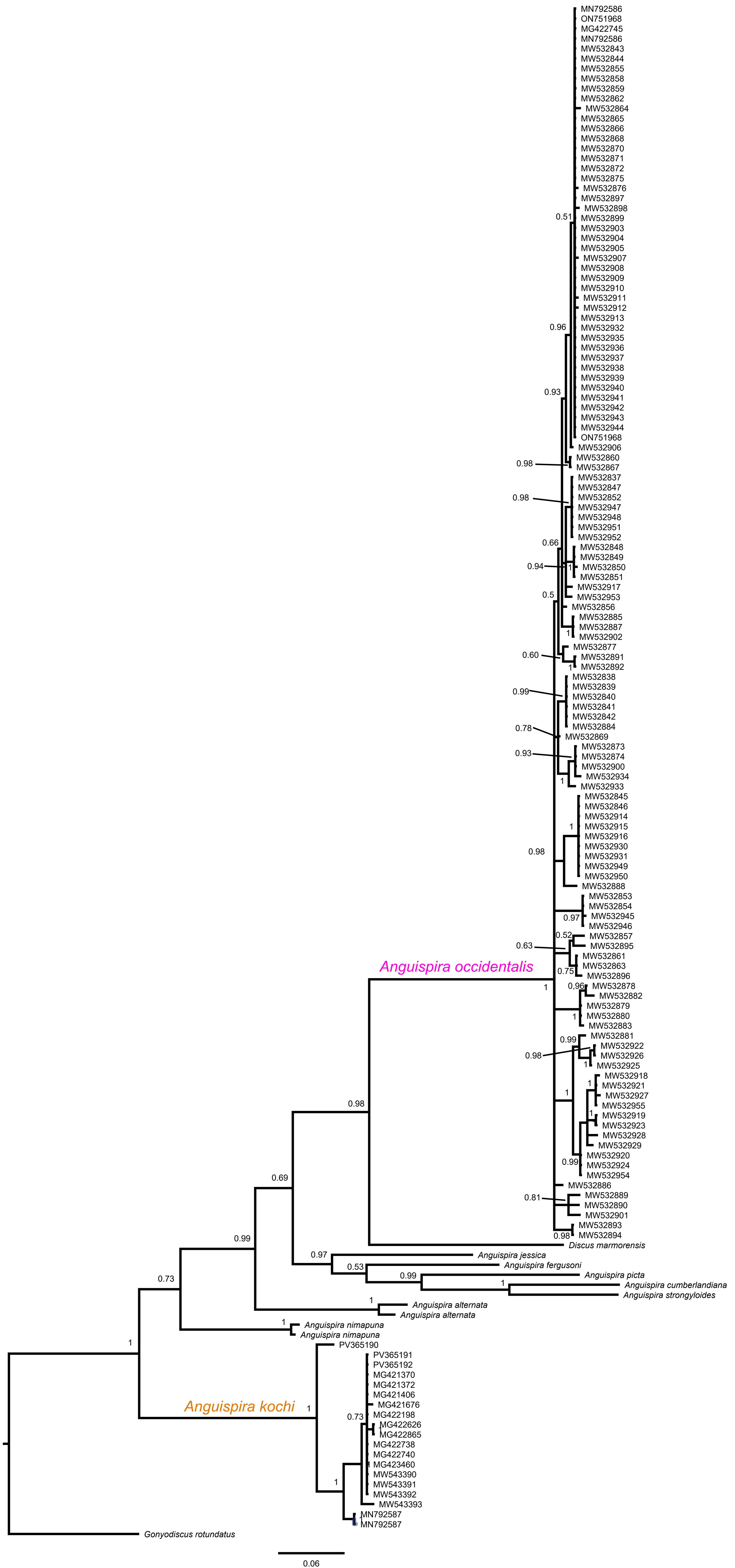

Supplement: Supplementary material 3 — Bayesian inference phylogenetic tree (50% majority-rule consensus) based on the COI barcoding marker [file zookeys-1261-241_article-171098__-s003.pdf]
